# Comparison of Stent-Assisted and Non-Stent-Assisted coil embolization in the treatment of Wide-Neck intracranial aneurysms: A Meta-Analysis

**DOI:** 10.1007/s10072-025-08409-y

**Published:** 2025-08-18

**Authors:** Yong-Lin He, Meng Ji, Zhi-Peng Liao, Rui Shang, Hai-Tao Hu, Yu-Hu Ma, Seidu A. Richard, Chang-Wei Zhang, Liang Niu

**Affiliations:** 1 Department of Neurosurgery, The Second Hospital of Lanzhou University, Lanzhou, China; 2 Department of Laboratory Medicine, Zhongnan Hospital of Wuhan University, Wuhan, China; 3 Department of Neurosurgery, West China Hospital, Sichuan University, Sichuan, China; 4 Department of Biochemistry and Forensic Sciences, School of Chemical and Biochemical Sciences, C. K. Tedam University of Technology and Applied Sciences (CKT-UTAS), Navrongo, UK 0215-5321 Ghana; 5 Institute of Neuroscience, Third Affiliated Hospital, Zhengzhou University, Zhengzhou, 450052 China

**Keywords:** Wide-neck intracranial aneurysms, Stent-assisted coiling, Coil embolization, Meta-analysis

## Abstract

**Objective:**

To evaluate the safety and efficacy of stent-assisted coiling (SAC) and non-stent-assisted coiling (NSAC) in the treatment of wide-neck intracranial aneurysms (WNAs).

**Methods:**

A meta-analysis was conducted to compare SAC and NSAC in treating WNAs. Primary outcomes were aneurysm occlusion rates and perioperative complication rates.

**Results:**

A total of 11 studies were included in this meta-analysis, encompassing 964 patients, with 547 assigned to the SAC group and 417 to the NSAC group. The SAC group demonstrated a significantly higher long-term complete occlusion rate (RR = 1.41, 95% CI [1.18, 1.68], *p* < 0.001) compared to the NSAC group. Additionally, the aneurysm recurrence rate was significantly lower in the SAC group (RR = 0.41, 95% CI [0.30, 0.56], *p* < 0.001). However, the SAC group exhibited a significantly higher incidence of ischemic complications (RR = 2.00, 95% CI [1.37, 2.94], *p* < 0.001), underscoring the increased risks associated with this treatment modality. When comparing the SAC group to the balloon-assisted coiling (BAC) and dual microcatheter coiling (DMC) groups, SAC demonstrated a significant advantage only in terms of recurrence rates: BAC (RR = 0.53, 95% CI [0.30, 0.92], *p* = 0.025) and DMC (RR = 0.49, 95% CI [0.30, 0.80], *p* = 0.004). In patients with ruptured aneurysms, the SAC group achieved a significantly higher complete occlusion rate (RR = 1.35, 95% CI [1.15, 1.59], *p* < 0.001) and a notably lower recurrence rate (RR = 0.29, 95% CI [0.15, 0.56], *p* < 0.001). Despite these positive outcomes, the overall complication risk (RR = 1.35, 95% CI [1.03, 1.78], *p* = 0.031) and the risk of ischemic complications (RR = 2.23, 95% CI [1.43, 3.46], *p* < 0.001) were significantly higher in the SAC group compared to the NSAC group.

**Conclusions:**

SAC provides superior long-term occlusion rates for RIA but is associated with higher perioperative ischemic complications than NSAC. Although it lowers recurrence compared to BAC and DMC, it may not be suitable for all patients.

## Introduction

Intracranial aneurysm (ICA) refers to an abnormal bulging lesion located on the intracranial arteries, most usually at the bifurcation. ICA has a high risk of rupture leading to spontaneous aneurysmal subarachnoid hemorrhage (aSAH) [1]. Also, aSAH has a high incidence and mortality rate worldwide [2, 3]. Nearly one-quarter of aSAH patients die and about half of the survivors are left with persistent neurological deficits [2, 3]. Wide-neck intracranial aneurysms (WNAs) are typically defined as aneurysms with necks widths greater than 4 millimeters or aneurysms with neck-to-base ratio greater than 1:2. Notably, conventional aneurysm embolization techniques may not effectively seal the neck, posing risks of coil displacement, thrombus formation, and other complications due to the wide neck [4–7]. Therefore, the treatment of WNAs is more challenging and often requires more complex methods such as stent-assisted coiling (SAC) [2, 8–10].

Although SAC has gradually been accepted in frontline clinical practice in recent years, there are still controversies regarding its safety. Studies have reported that the complication rate and mortality rate are significantly higher when SAC is used to treat ruptured intracranial aneurysms (RIA) compared to unruptured intracranial aneurysms (UIA) [11–14]. The hypercoagulable state in the acute phase of SAH can induce thrombosis within the stent, making dual antiplatelet therapy necessary in SAC to minimize thromboembolic complications [15–17]. However, the use of dual antiplatelet drugs can not only cause aneurysm rebleeding and increase the incidence of hemorrhagic complications, but it can also complicate surgical procedures during the acute phase of aSAH [18–20]. Another study showed that in RIA, the overall perioperative incidence of thromboembolic and hemorrhagic complications with SAC was as high as 20.2%[21]. Therefore, the safety of SAC treatment still poses significant risks. However, in contrast, multiple studies have shown that the perioperative complication rates between SAC and traditional coil embolization are comparable [21, 22]. A recent meta-analysis also showed that SAC achieved a better complete occlusion rate and a lower recurrence rate during follow-up [23]. This suggests that SAC may have greater advantages in treating WNAs.

We found that current research lacks proper and objective evaluations of the safety and efficacy of SAC in treating WNAs. Therefore, we conducted a systematic review and meta-analysis with data from high-quality studies to assess the safety and efficacy of SAC versus non-stent-assisted coil embolization (NSAC) for WNAs.

## Methods and materials

### Protocol and registration

This systematic review and meta-analysis compared the efficacy and safety of SAC to NSAC for the treatment of WNAs. The study adhered strictly to PRISMA (Preferred Reporting Items for Systematic Reviews and Meta-Analyses) guidelines [24]. The data used in this study were derived from previously published literature thus, ethical approval or informed patient consent are not required.

### Eligibility criteria

This meta-analysis included studies that met the following criteria: (1) randomized controlled trials (RCTs) or observational cohort studies comparing SAC to NSAC for the treatment of WNAs. (2) studies involving patients diagnosed with WNAs using digital subtraction angiography (DSA). (3) studies reporting primary efficacy outcomes, such as complete aneurysm occlusion rate, and safety outcomes, including procedural complications. (4) a minimum sample size of 10 patients per group. (5) preference given to matched data in studies employing propensity score matching (PSM).

Exclusion criteria included: (1) studies that did not explicitly specify the inclusion of patients with WNAs. (2) studies lacking sufficient outcome data for comparison between groups. (3) non-English language publications. (4) reviews, case reports, expert opinions, conference abstracts, commentaries, letters, and animal studies, and (5) duplicate publications, with only the most recent or comprehensive version retained.

### Search and study selection

In this meta-analysis, published studies related to SAC and NSAC treatments of WNAs were systematically searched in PubMed, EMBASE, Cochrane Library, and Web of Science databases from the inception of these databases until July 31, 2024. The search strategy employed keywords such as “wide-neck intracranial aneurysm,” “stent-assisted,” “non-stent-assisted,” and “coil embolization.” Additionally, manual searches of the reference lists from relevant studies were conducted to ensure comprehensive inclusion of all eligible research papers.

Two independent reviewers screened the titles and abstracts to exclude studies that are not aligned with the research aim and objectives after the removal of duplicate records. Full-text reviews were conducted to confirm eligibility, with detailed documentation and justification for any exclusions for the remaining studies. Discrepancies between reviewers were resolved through discussion; if consensus could not be achieved, a third reviewer was consulted to provide a final decision throughout this process.

### Data collection

Data extraction was independently conducted by two researchers, covering fundamental information of the included studies such as authors, publication year, and country as well as patient characteristics such as age, gender, aneurysm size and location, and rupture status. Also, detailed information on interventions, and key efficacy and safety outcomes were extracted. The primary outcomes included aneurysm occlusion rate, aneurysm recurrence rate, and the incidence of perioperative complications. Secondary outcomes included perioperative mortality rate, aneurysm retreatment rate, and favorable clinical outcomes, defined as a modified Rankin Scale (mRS) score of 0–2 or a Glasgow Outcome Scale (GOS) score of 4–5. Furthermore, data from matched groups were prioritized for extraction for studies that performed PSM analysis. All data were cross-checked after extraction to ensure accuracy and consistency. In case of discrepancies during the extraction process, the researchers resolved them through discussion, and a third-party researcher was consulted for arbitration if necessary.

### Risk of bias

The risk of bias in the included studies was independently evaluated by two researchers. The assessments were conducted according to the criteria outlined in the Cochrane Handbook for Systematic Reviews of Interventions for RCTs [25]. The following domains were evaluated: random sequence generation, allocation concealment, blinding of participants and personnel, blinding of outcome assessment, incomplete outcome data, selective reporting, and other relevant biases. Each domain in the included studies was classified as having a low, high, or unclear risk of bias, with supporting information provided from the study reports. For retrospective cohort studies, the Newcastle-Ottawa Scale (NOS) was used to evaluate selection bias, information bias, and the control of confounding factors [26]. The NOS assigns a score ranging from 0 to 9, with a score of ≥ 7 indicating a study with a low risk of bias. Any disagreements between the two researchers were resolved through discussion, and if necessary, a third researcher was consulted to make the final determination.

### Statistical analysis

All statistical analyses were performed using Stata 16.0. Binary outcomes were expressed as risk ratios (RR), and continuous outcomes as mean differences (MD), both accompanied by 95% confidence intervals (CI). Results were visualized with forest plots. Heterogeneity was assessed using the I² statistic; a fixed-effects model was applied when I² was ≤ 50%, and a random-effects model (DerSimonian and Laird method) was employed when I² exceeded 50%. Subgroup analyses were performed based on aneurysm rupture status and type of intervention. Publication bias was evaluated using funnel plots and further tested with Egger’s test and Harbord’s test. All p-values were two-sided, with *p* < 0.05 considered statistically significant.

## Results

### Characteristics of included studies

**Fig. 1:**
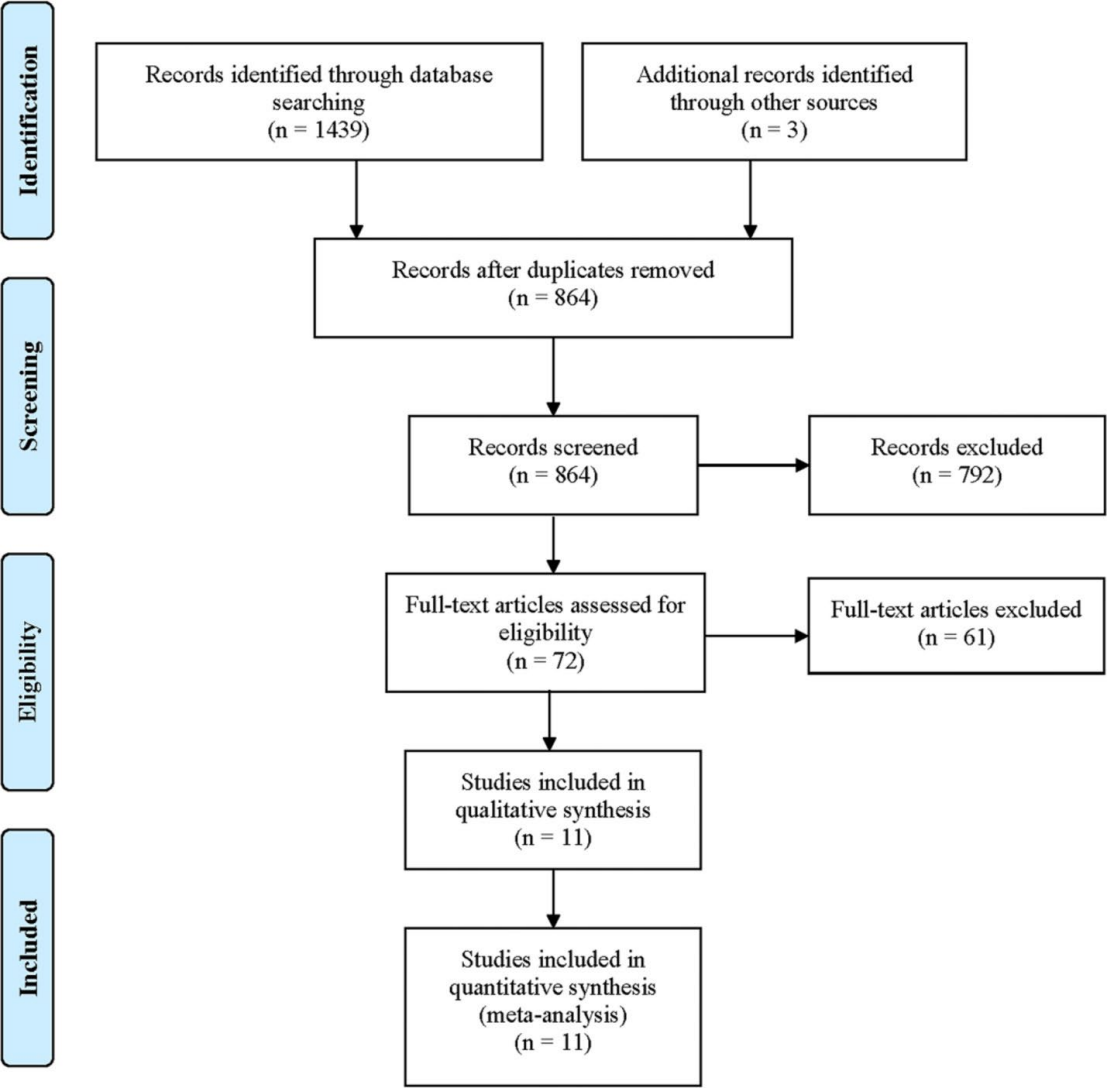
Flowchart of study selection for the present study

A comprehensive literature search yielded 1,442 relevant articles. After removing duplicates, 864 studies underwent preliminary screening. Also, 792 articles were excluded for not aligning with the study objectives following a detailed review of titles and abstracts. Full-text assessments were conducted on the remaining 72 studies, resulting in the exclusion of 61 articles. Ultimately, 11 studies [27–37] were included in this systematic review and meta-analysis **(**Fig. 1**)**. These studies primarily focused on comparing the efficacy of SAC with NSAC, including conventional coiling (CA), balloon-assisted coiling (BAC), and dual microcatheter coiling (DMC). Notably, four studies provided comparative data between two of these treatment groups. In terms of patient characteristics, four studies concentrated on RIA, three studies focused on UIA, while the remaining four did not explicitly differentiate between aneurysm types. The SAC group employed a variety of stents, including Solitaire, Enterprise, and Neuroform stents, while the balloon devices used in BAC were predominantly Hyperglide, Hyperform, and Scepter C. The follow-up period across studies varied significantly, ranging from 3 months to 58.36 months. All included studies utilized a retrospective design, with one study employing a PSM approach for data analysis. The definition of WNAs varied slightly among the studies, with most defining wide-neck as a neck diameter ≥ 4 mm or a dome-to-neck ratio < 2. The baseline characteristics of the included studies are detailed in Tables 1 and 2.

**Table 1:** Summary of studies selected for systematic review and meta-analysis

SAC/ NSAC(n)	Patients	Comparison	Stent brand	Balloon brand	Study period	Follow-up (month)	NOS
133/289	RIA	SAC versus CA	NR		2012.1-2014.12	36[3-59]	8
136/136	RIA	SAC versus CA	Solitaire	NR	2018.1-2022.5	12	9
122/164	UIA	SAC versus BAC	Enterprise	Hyperglide Hyperform	NR		8
			Solitaire				
			Neuroform				
69/32	unclassified	SAC versus BAC	Neuroform: 49	Hyperglide: 30	2009-2010	10.7±7.5	9
			Enterprise: 20	Hyperform: 2			
65/32	RIA	SAC versus BAC	NR		2011.11-2014.12	14.6[5-43]	8
71/35	UIA	SAC versus BAC	Neuroform	Hyperglide	2008-2011	12	9
			Enterprise	Hyperform			
35/39	unclassified	SAC versus CA	NR		2003.1-2016.10	58.36±25.08	8
35/34		SAC versus DMC					
127/35	unclassified	SAC versus BAC	Neuroform	Hyperglide Hyperform Scepter C	2008.9-2013.12	19.5[0.5-49]	9
127/45		SAC versus DMC	Enterprise				
44/30	RIA	SAC versus BAC	NR		2016.9-2020.12	6	8
44/11		SAC versus CA					
45/28	unclassified	SAC versus BAC	NR		2008.1-2012.6	3	7
45/20		SAC versus DMC					
120/60	UIA	SAC versus DMC	Neuroform	NR	2006-2012	24.3±21.6	9
			Enterprise				

*USA *United States of America; *RS *retrospective; *PSM *propensity matching analysis; *SAC *stent-assisted coiling; *NSAC *non-stent-assisted coiling; *RIA *ruptured intracranial aneurysm; *UIA *unruptured intracranial aneurysm; *CA *coiling alone; *BAC *balloon-assisted coiling; *DMC *dual microcatheter coiling; *NR *not reported; *NOS *Newcastle-Ottawa Scale

**Table 2:** Baseline characteristics of included studies

Author	Groups	Age(years)	Women	Aneurysm size(mm)	Aneurysm neck(mm)	Dome-to-neck ratio	Definition of Wide-Neck
Zuo Q.	SAC	58.1±11.7	93	5.5±3.2	NR		NR
	CA	56.2±12.1	182	5.0±2.5			
Chen ZW.	SAC	61.41±9.37	93	5.00[4.00–6.00]	4.50[4.00, 5.08]	1.18[1.00, 1.30]	NR
	CA	60.54±9.70	95	5.00[4.00–6.00]	4.00[3.00, 4.50]	1.25[1.19, 1.43]	
Consoli A.	SAC	NR	80	NR			either as a neck >4 mm or a dome-to-neck aspect ratio <1.5, or both
	BAC		108				
Chalouhi N.	SAC	54.2	57	6.8	NR		with a fundus-to-neck ratio of <2 or a neck diameter of d ≥4 mm
	BAC	53.7	26	6.4			
Cai KF.	SAC	56.6±11.7	49	5.1±2.2	4.0±1.4	1.2±0.3	with a fundus/neck ratio of <2 or a neck diameter of ≥4 mm
	BAC	56.5±10.9	22	5.6±2.2	3.4±1.0	1.3±0.2	
Peterson E.	SAC	NR				1.18[1.01-1.59]	NR
	BAC					1.5[1.16-1.66]	
Kim HS.	SAC	55.64±10.04	82	NR			the neck was larger than 4 mm or the dome-to-neck ratio was less than 2
	CA						
	DMC						
Chung EJ.	SAC	55.45±11.849	108	6.65±3.347	4.95±2.092	1.16±0.366	either a wide neck (≥4 mm) or a dome-to-neck ratio <2
	BAC	51.45±12.043	32	6.22±2.228	3.79±0.987	1.46±0.387	
	DMC	62.00±10.302	32	9.89±4.351	5.50±2.555	1.68±0.568	
Onay M.	SAC	NR			4.52±1.56	1.94±0.36	with a dome-neck ratio <2 or a neck diameter >4 mm
	BAC				3.66±1.32	1.55±0.41	
	CA				3.11±1.31	1.52±0.26	
Pan JW.	SAC.BAC.DMC	NR					with neck ≥4 mm or dome/neck ratio ≤1.2
Starke RM.	SAC	56.7±11.3	97	8.2±4.4	4.8±1.7	NR	as a dome to neck ratio of<2 or a neck diameter >4 mm
	DMC	57.9±11.1	43	8.2±4.1	4.6±1.3		

*SAC *stent-assisted coiling; *CA *coiling alone; *BAC *balloon-assisted coiling; *DMC *dual microcatheter coiling; *NR *not reported

### Analysis of outcomes

**Fig. 2:**
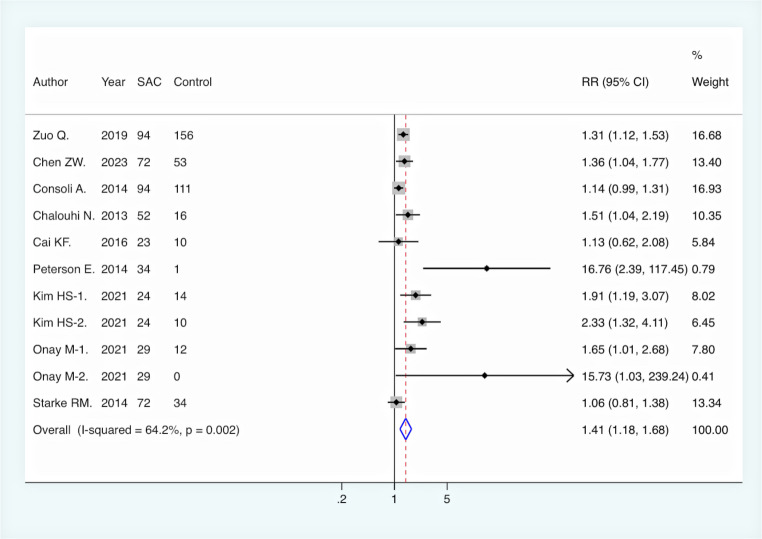
Comparison of complete occlusion rate at last clinical follow-up between SAC and NSAC groups

**Fig. 3:**
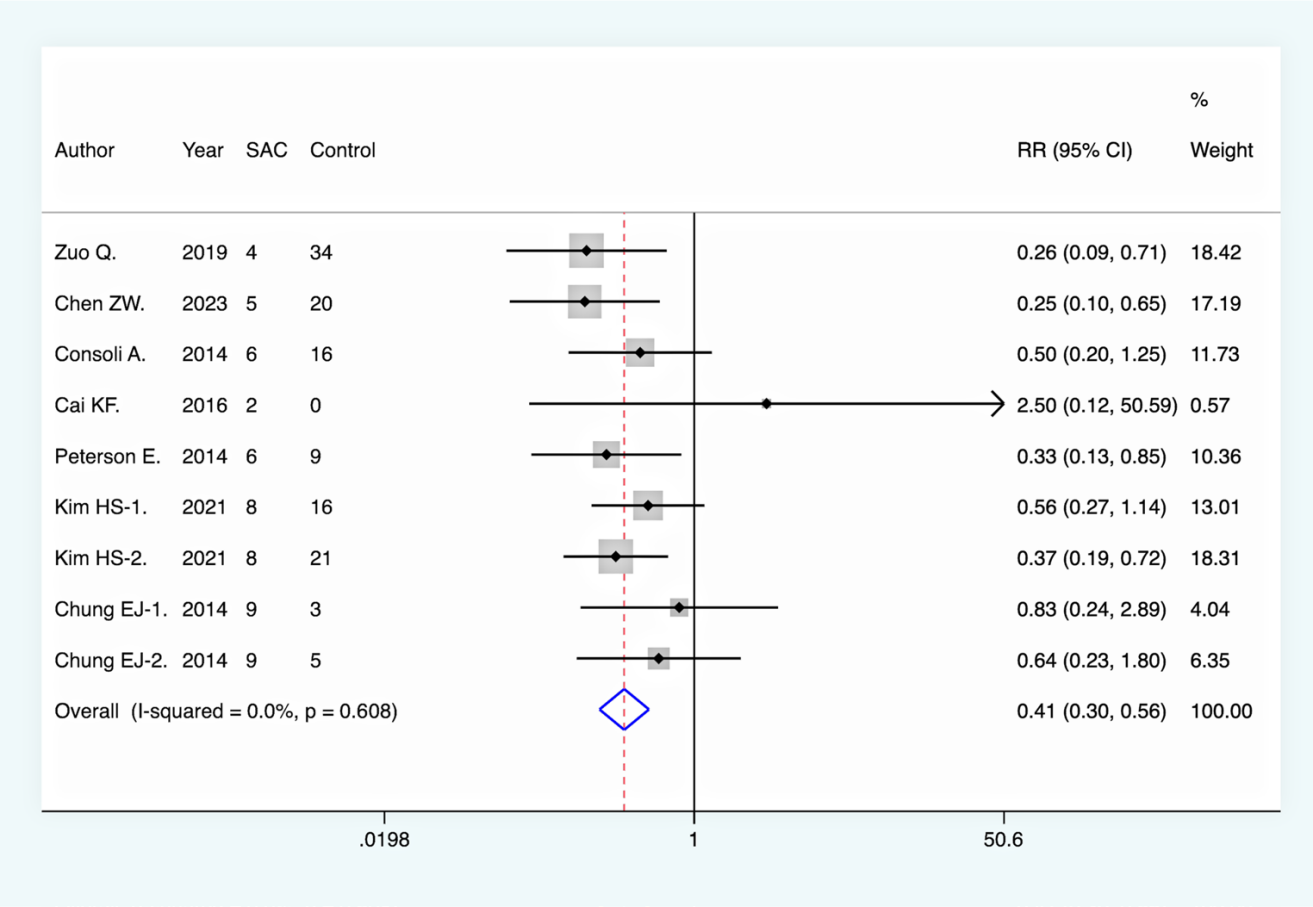
Comparison of recurrence rate between the two groups

**Fig. 4:**
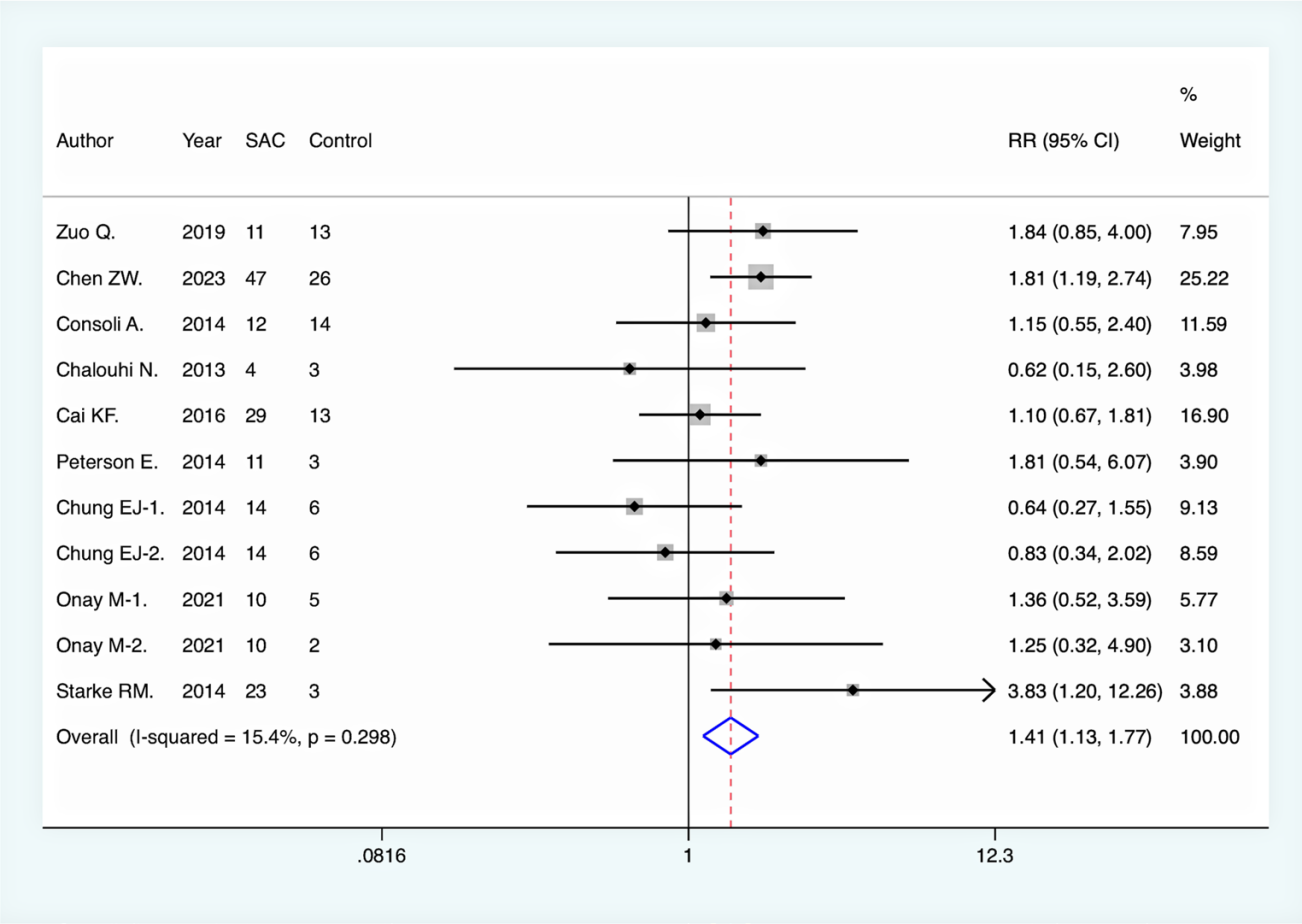
Comparison of total complication rate between the two groups

**Fig. 5:**
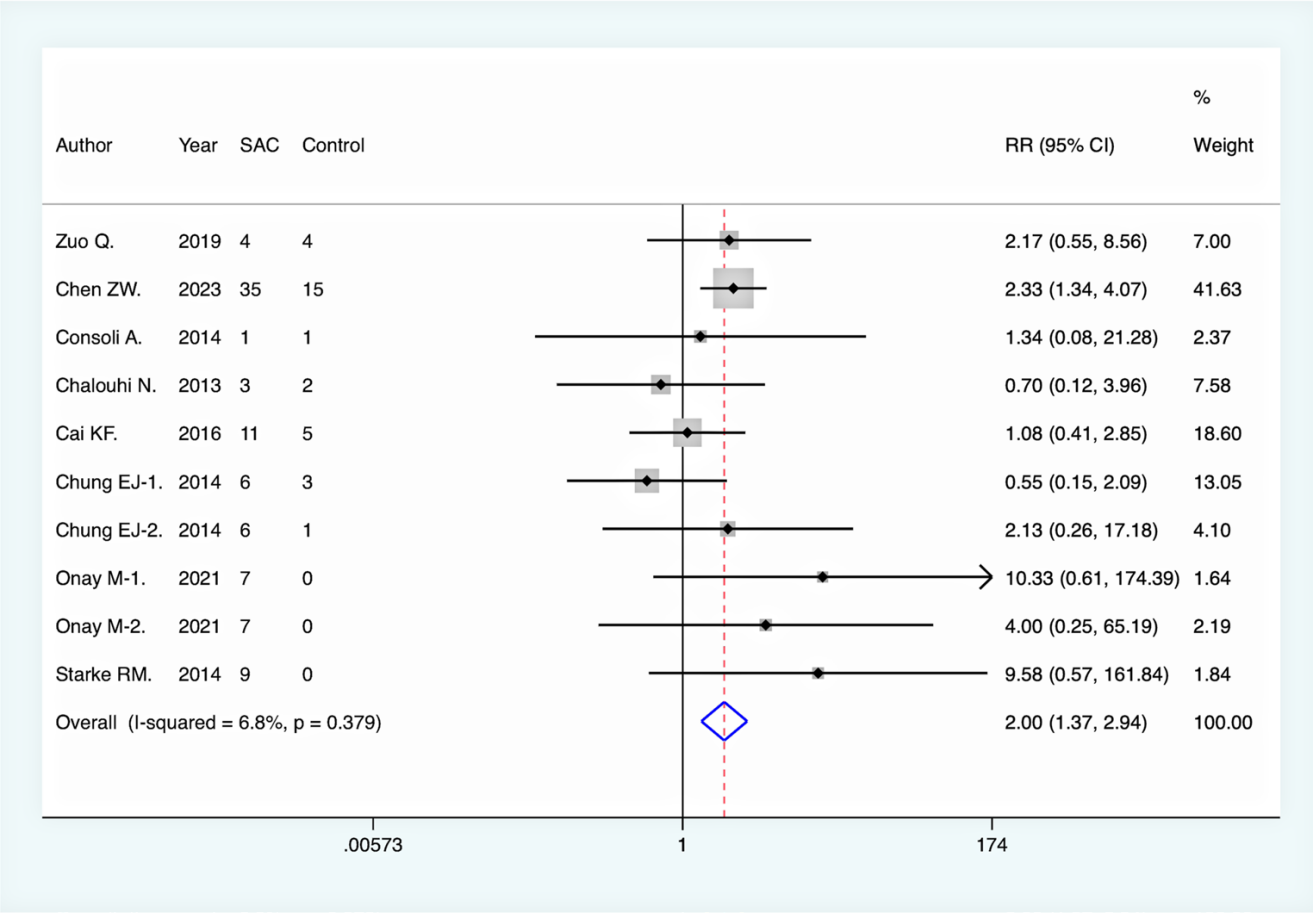
Comparison of ischemic complication rate between SAC and NSAC groups

All included studies reported immediate complete aneurysm occlusion rates following endovascular intervention. Pooled analysis revealed no significant difference between the SAC group and NSAC group in terms of immediate complete occlusion (RR = 1.00, 95% CI [0.89, 1.12], *p* = 0.948). However, at the final DSA follow-up, the rate of complete aneurysm occlusion was significantly higher in the SAC group compared to the NSAC group (RR = 1.41, 95% CI [1.18, 1.68], *p* < 0.001), suggesting a long-term efficacy advantage of SAC **(**Fig. 2**).** Moreover, the aneurysm recurrence rate was significantly lower in the SAC group (RR = 0.41, 95% CI [0.30, 0.56], *p* < 0.001), further supporting its effectiveness in reducing recurrence risk **(**Fig. 3**).** On the other hand, the overall periprocedural complication rate was higher in the SAC group (RR = 1.41, 95% CI [1.13, 1.77], *p* = 0.003) **(**Fig. 4**)**, with a notably increased incidence of ischemic complications (RR = 2.00, 95% CI [1.37, 2.94], *p* < 0.001) **(**Fig. 5**)**, indicating an elevated risk associated with this intervention. There was no significant difference between the two groups in terms of hemorrhagic complications (RR = 0.93, 95% CI [0.63, 1.38], *p* = 0.730).

Among the secondary outcomes, the SAC group exhibited a trend towards reduced retreatment rates (RR = 0.88, 95% CI [0.55, 1.40], *p* = 0.579) and coil protrusion rates (RR = 0.65, 95% CI [0.29, 1.44], *p* = 0.289), although these differences did not reach statistical significance. Furthermore, no significant differences were observed between the SAC and NSAC groups in terms of periprocedural mortality (RR = 1.03, 95% CI [0.49, 2.15], *p* = 0.944), as well as favorable outcomes at discharge (RR = 1.06, 95% CI [0.91, 1.23], *p* = 0.911) or at final follow-up (RR = 0.99, 95% CI [0.95, 1.04], *p* = 0.715). The detailed outcome is summarized in Table 3.

**Table 3:** Pooled results of primary and secondary outcomes of the meta-analysis

Outcomes	No. of datas	Statistical method	Pooled results				Heterogeneity	P value	P value for Egger's test	P value for harbord's test
			RR	LCI	UCI	P value	I^2^(%)			
Immediate complete occlusion rate	13	random	1.00	0.89	1.12	0.948	54.6	0.009	0.165	0.190
Complete occlusion rate at last clinical follow-up	11	random	1.41	1.18	1.68	<0.001	64.2	0.002	0.007	0.031
Recurrence rate	9	fixed	0.41	0.30	0.56	<0.001	0	0.608	0.096	0.046
Retreatment rate	5	fixed	0.88	0.55	1.40	0.579	33	0.202	0.804	0.772
Perioperative mortality rate	7	fixed	1.03	0.49	2.15	0.944	0	0695	0.544	0.535
Total complication rate	11	fixed	1.41	1.13	1.77	0.003	15.4	0.298	0.331	0.070
Hemorrhagic complication rate	10	fixed	0.93	0.63	1.38	0.730	0	0.729	0.090	0.045
Ischemic complication rate	10	fixed	2.00	1.37	2.94	<0.001	6.8	0.379	0.942	0.532
Coil protrusion rate	7	fixed	0.65	0.29	1.44	0.289	34.7	0.190	0.068	0.142
Favorable outcomes at discharge	4	fixed	1.00	0.95	1.06	0.911	0	0.601	0.511	0.492
Favorable outcomes at last clinical follow-up	8	fixed	0.99	0.95	1.04	0.715	0	0.862	0.267	0.218

*RR *risk ratios; *LCI *lower confidence interval; *UCI *upper confidence interval

### Analysis of subgroup outcomes

Given that the NSAC group comprised three distinct treatment modalities such as CA, BAC, and DMC, we performed a subgroup analysis to further explore the differences in primary outcomes based on the type of control treatment. Compared to the CA group, the SAC group demonstrated a significantly higher rate of complete aneurysm occlusion at the final follow-up (RR = 1.45, 95% CI [1.13, 1.85], *p* = 0.003) and a markedly lower recurrence rate (RR = 0.33, 95% CI [0.20, 0.56], *p* < 0.001). However, the SAC group was associated with significantly higher rates of overall complications (RR = 1.77, 95% CI [1.24, 2.52], *p* = 0.002) and ischemic complications (RR = 2.37, 95% CI [1.42, 3.97], *p* = 0.001) compared to the CA group. When compared to the BAC and DMC groups, the SAC group exhibited a trend toward reduced recurrence rates (BAC: RR = 0.53, 95% CI [0.30, 0.92], *p* = 0.025; DMC: RR = 0.49, 95% CI [0.30, 0.80], *p* = 0.004), though no significant differences were observed for other primary outcomes **(**Table 4**).**

**Table 4:** Subgroup analysis of primary outcomes based on the type of control treatment

Subgroup outcomes		No. of datas	Pooled results				Heterogeneity	
			RR	LCI	UCI	P value	I^2^(%)	P value
Immediate complete occlusion rate	SAC versus CA	3	1.08	0.97	1.21	0.175	0	0.483
	SAC versus BAC	6	0.87	0.68	1.10	0.247	70.2	0.005
	SAC versus DMC	4	1.02	0.75	1.39	0.897	69.0	0.021
Complete occlusion rate at last clinical follow-up	SAC versus CA	4	1.45	1.13	1.85	0.003	50.3	0.110
	SAC versus BAC	5	1.45	0.99	2.13	0.056	72.7	0.005
	SAC versus DMC	2	1.51	0.69	3.30	0.303	84.2	0.012
Recurrence rate	SAC versus CA	3	0.33	0.20	0.56	<0.001	22.6	0.275
	SAC versus BAC	4	0.53	0.30	0.92	0.025	0	0.478
	SAC versus DMC	2	0.44	0.25	0.77	0.004	0	0.386
Total complication rate	SAC versus CA	3	1.77	1.24	2.52	0.002	0	0.875
	SAC versus BAC	6	1.08	0.77	1.50	0.663	0	0.722
	SAC versus DMC	2	1.76	0.89	3.48	0.103	77.6	0.034
Hemorrhagic complication rate	SAC versus CA	3	1.25	0.70	2.21	0.446	26.2	0.258
	SAC versus BAC	5	0.79	0.42	1.47	0.457	0	0.920
	SAC versus DMC	2	0.57	0.20	1.64	0.298	0	0.402
Ischemic complication rate	SAC versus CA	3	2.38	1.43	3.97	0.001	0	0.925
	SAC versus BAC	5	1.22	0.65	2.30	0.539	0.6	0.403
	SAC versus DMC	2	4.44	0.87	22.65	0.073	0	0.383

*SAC *stent-assisted coiling; *CA *coiling alone; *BAC *balloon-assisted coiling; *DMC *dual microcatheter coiling; *RR *risk ratios; *LCI *lower confidence interval; *UCI *upper confidence interval

We also conducted a subgroup analysis based on aneurysm rupture status. In patients with RIA, the SAC group demonstrated a significantly higher complete occlusion rate at final follow-up (RR = 1.35, 95% CI [1.15, 1.59], *p* < 0.001) and a significantly lower recurrence rate (RR = 0.29, 95% CI [0.15, 0.56], *p* < 0.001) compared to the control group. However, the SAC group had a higher risk of overall complications (RR = 1.35, 95% CI [1.03, 1.78], *p* = 0.031) and ischemic complications (RR = 2.23, 95% CI [1.43, 3.46], *p* < 0.001). In patients with UIA, no significant differences were found between the SAC and NSAC groups in the primary outcomes, except for a significantly lower recurrence rate in the SAC group (RR = 0.42, 95% CI [0.22, 0.81], *p* = 0.010). While the SAC group showed a trend toward higher ischemic complications, the difference did not reach statistical significance (RR = 4.95, 95% CI [0.74, 33.19], *p* = 0.099). In patients with unclassified aneurysms, no significant differences in overall outcomes were observed between the SAC and NSAC groups, except for a significant advantage of reduction in recurrence in the SAC group (RR = 0.51, 95% CI [0.34, 0.78], *p* = 0.002) **(**Table 5**)**. In summary, SAC demonstrated clear efficacy advantages in specific patient populations, particularly in reducing recurrence rates. However, this benefit was accompanied by a higher risk of complications, especially in patients with RIA.

**Table 5:** Subgroup analysis of primary outcomes based on aneurysm rupture status

Subgroup outcomes		No. of datas	Pooled results				Heterogeneity	
			RR	LCI	UCI	P value	I^2^(%)	P value
Immediate complete occlusion rate	RIA	3	1.02	0.89	1.18	0.761	24.1	0.268
	UIA	3	1.04	0.87	1.23	0.691	35.7	0.211
	unclassified	7	0.97	0.75	1.26	0.832	70.1	0.003
Complete occlusion rate at last clinical follow-up	RIA	5	1.35	1.15	1.59	<0.001	15.5	0.316
	UIA	3	1.25	0.80	1.96	0.321	80.8	0.006
	unclassified	3	1.77	1.37	2.30	<0.001	0	0.418
Recurrence rate	RIA	3	0.29	0.15	0.56	<0.001	5.7	0.346
	UIA	2	0.42	0.22	0.81	0.010	0	0.521
	unclassified	4	0.51	0.34	0.78	0.002	0	0.635
Total complication rate	RIA	5	1.54	1.16	2.03	0.002	0	0.613
	UIA	3	1.82	1.06	3.14	0.031	34.8	0.216
	unclassified	3	0.71	0.40	1.26	0.245	0	0.907
Hemorrhagic complication rate	RIA	5	1.14	0.70	1.86	0.594	0	0.423
	UIA	2	0.62	0.21	1.84	0.387	0	0.381
	unclassified	3	0.69	0.31	1.56	0.373	0	0.955
Ischemic complication rate	RIA	5	2.23	1.43	3.46	<0.001	0	0.484
	UIA	2	4.95	0.74	33.19	0.099	6.1	0.302
	unclassified	3	0.86	0.34	2.15	0.741	0	0.548

*RIA *ruptured intracranial aneurysm; *UIA *unruptured intracranial aneurysm; *RR *risk ratios; *LCI *lower confidence interval; *UCI *upper confidence interval

### Publication bias

Publication bias was systematically evaluated for all outcome measures using the Egger and Harbord tests. The analyses revealed that only the complete occlusion rate at the last clinical follow-up exhibited potential publication bias, suggesting that published studies may favor positive outcomes in this metric. In contrast, the remaining outcome measures demonstrated a low risk of publication bias, indicating robustness in the available evidence. All results are summarized in Table 3.

## Discussion

This systematic review and meta-analysis not only provide important insights into the efficacy and safety of SAC compared to NSAC, but also revealed the applicability and potential risks of SAC in different patient groups. The results show that SAC had a significantly higher complete occlusion rate and significantly lower recurrence rate during long-term follow-up compared to the NSAC group, indicating that SAC has a clear advantage in preventing aneurysm recurrence in the long term. The potential mechanism may be attributed to the physical support provided by the stent at the aneurysm neck, which helps to maintain the stability of the embolization coils, reducing the likelihood of coil displacement or migration, thereby improving the durability of the occlusion.

Notably, the risk of perioperative complications in the short term cannot be ignored, particularly the incidence of ischemic events although SAC demonstrates outstanding long-term efficacy. Compared to the NSAC group, the overall complication rate in the SAC group was significantly increased, especially in comparison with the CA group. This result may be related to the introduction of foreign material into the vascular system during stent placement, which interferes with local hemodynamics and induces platelet aggregation, thereby increasing the risk of thrombosis. It was observed that the incidence of postoperative ischemic stroke was as high as 20.6% (7/34), which is consistent with our findings [38]. Therefore, SAC usually requires long-term antiplatelet therapy to reduce this risk [39]. Multiple studies suggested that postoperative dual antiplatelet therapy is effective in preventing the occurrence of ischemic stroke in most cases [38, 40]. However, this also brings an additional risk of bleeding, especially in patients with contraindications to antiplatelet drugs or those with other bleeding tendencies [41–43]. Therefore, for high-risk patients such as those with a history of thrombosis or severe arteriosclerosis, the application of SAC should be carefully considered. Future research should focus on effectively managing short-term complications while pursuing long-term efficacy, particularly in controlling perioperative risks [2, 44].

Subgroup analysis revealed significant differences in the effectiveness of SAC in patients with different types of intracranial aneurysms. Also, SAC demonstrated more significant efficacy in improving complete occlusion rates and reducing recurrence rates for patients with RIA, but this was also accompanied by a higher risk of perioperative ischemic complications. A study suggested that patients with a higher degree of bleeding have a greater risk of developing ischemic stroke and/or hemorrhagic complications compared to those with a lower degree of bleeding which also supports our conclusions [45]. These patients have increased treatment difficulty due to the fragility of their arterial wall structure and pathological complexity, making them more prone to stent implantation-related complications during surgery, such as displacement of embolic materials or intravascular injury [37]. Therefore, for these high-risk patients, although SAC can significantly improve long-term efficacy, managing short-term risks remains crucial, especially in optimizing strategies for perioperative vascular protection and postoperative complication management. In patients with UIA, although the incidence of ischemic events in the SAC group increased, it did not reach statistical significance, suggesting that its safety in such patients is relatively ideal. Another study also suggested that the complication rate of stent-assisted coiling was 10 times higher in ruptured aneurysms compared to unruptured aneurysms [46]. It also pointed out that stent-assisted coiling in ruptured aneurysms was associated with increased morbidity and mortality, which aligns with our view [46]. However, the choice between SAC and other treatment options still requires a comprehensive evaluation based on the individual patient’s anatomy, medical history, and other factors to develop the most suitable treatment strategy.

Notably, there were no statistically significant differences in other major outcome measures although SAC had a significantly lower recurrence rate compared to BAC and DMC, indicating that SAC may not be the best choice for all types of wide-neck aneurysms. Also, BAC and DMC have shown effectiveness in certain clinical settings, with these techniques generally being simpler to perform and causing less vascular interference, potentially leading to fewer postoperative complications and faster recovery times [28]. Specifically, BAC provides immediate arterial wall support during embolization, significantly reducing the risk of embolic material dislodgement and minimizing interference with hemodynamics [47, 48]. The DMC technique, through the use of dual microcatheters, enhances guiding capability and improves the precision of embolization, indicating its efficacy in treating small and micro aneurysms. Therefore, when evaluating different patients and the characteristics of their aneurysms, it is necessary to choose the appropriate treatment option based on specific conditions. For example, DMC is more suitable for aneurysms with a dome-to-neck ratio of 1.1–1.2, whereas stent treatment is better for proximal aneurysms with a very wide neck ≥ 7 mm and poor vascular conditions, such as funnel-shaped aneurysms with a dome-to-neck ratio < 1.0. For emergency cases, both BAC and DMC are superior to SAC [49, 50]. Therefore, in certain cases, BAC and DMC may serve as effective alternatives to SAC [28].

Currently, for the interventional treatment of WNAs, emerging technologies such as flow diverters (FD) and woven endobridge devices (WEB) are gradually showing potential clinical applications, in addition to traditional embolization techniques [51–54]. FD work by altering the hemodynamics at the aneurysm neck, promoting intra-aneurysmal thrombosis to achieve long-term occlusion [55]. Compared to SAC, its advantage lies in not requiring direct entry into the aneurysm sac for embolization, thus reducing the risk of complications such as embolic material dislodgement or migration [56]. The WEB device, designed specifically for complex wide-neck aneurysms, is a mesh structure that forms a double-layer occlusion inside the aneurysm sac, reducing direct interference with the aneurysm neck and surrounding vessels [57]. In terms of perioperative complication risk, the WEB device showed certain advantages, particularly in reducing the incidence of intraoperative and postoperative ischemic complications [58]. However, the long-term efficacy of the WEB device remains controversial. A single-center study indicated that its recurrence rate and complete occlusion rate were lower than SAC, but this study involved only a moderate number of patients and lacked long-term follow-up data for the WEB group, with a shorter follow-up duration than the SAC group [9]. Also, a recent systematic review suggested that although the overall complication and mortality rates were very low, thromboembolic complications remain a major issue [59]. Additionally, another systematic review indicated that approximately 10% of patients treated with the WEB device for WNAs underwent retreatment, and the risk factors for aneurysm recurrence remain unclear, requiring further research [60]. A recent meta-analysis also suggested that, compared to SAC, the WEB group had lower rates of complete and adequate immediate occlusion, with a similar retreatment rate and no significant difference in neurological complications [58]. Therefore, the efficacy of WEB treatment may require further investigation.

With the continuous advancement of neurointerventional technologies, the treatment of WNAs is gradually becoming more individualized and precise. This study provides strong support for the efficacy and safety of SAC, but future treatments should not remain focused solely on comparing current methods. True progress may come from the integration of multidisciplinary technologies and the development of AI-assisted precision treatment models. These innovations are expected to help doctors develop more optimized treatment plans for each patient through real-time monitoring and individualized risk prediction, thus improving efficacy and reducing complications. In the future, comprehensive evaluations of vascular structures and biomechanics will offer more forward-looking, personalized solutions for the treatment of WNAs and thus, further improving patient outcomes.

## Limitations

This study still has some limitations. Firstly, the follow-up times across studies are inconsistent, and short-term follow-up in some studies are insufficient to fully assess long-term recurrence rates and complete occlusion rates, which limits conclusions on long-term efficacy. Secondly, there are currently lack of studies comparing technologies such as FD and WEB with SAC in clinical practice, resulting in a shortage of high-level evidence. Thirdly, Lastly, all included studies were retrospective cohort studies, with a high risk of selection bias.

Meanwhile, It is also important to note that the majority of SAC procedures included in this meta-analysis were performed using laser-cut stents. This predominance was not due to selective inclusion but instead reflects the current distribution of comparative evidence in the published literature. During study design and article screening, we did not restrict inclusion based on stent structure; however, studies that explicitly involved braided stents and directly compared them with NSAC were extremely limited or absent. In contrast, laser-cut stents such as Neuroform and Enterprise have been more widely used in prospective clinical trials, especially for wide-neck aneurysms, making them more frequently represented in eligible studies.

Moreover, we observed that approximately half of the included studies did not report the specific stent type (i.e., laser-cut vs. braided), which further limited the feasibility of performing stent-type-specific subgroup analysis. While this ambiguity restricts our ability to draw conclusions about specific device subtypes, it also reflects real-world practice, where such details are not always consistently reported.

Although a recent retrospective study[53] suggested no significant difference in clinical outcomes between laser-cut and braided stents, these findings have yet to be confirmed in prospective, directly comparative trials. Therefore, we recommend that future studies not only focus more on the comparative performance of braided stents, but also clearly document the type and structural characteristics of the devices used. These steps are essential for improving the granularity, comparability, and generalizability of evidence in this evolving field.

Lastly, it is worth noting that many of the studies included in this meta-analysis utilized earlier-generation devices or techniques, such as conventional CA, BAC, and DMC. Although these non-stent-assisted approaches played an important role historically, they are now less commonly used due to the development and widespread adoption of SAC, especially for wide-neck intracranial aneurysms. The evolution of endovascular devices, including more flexible, low-profile, and flow-diverting stents, has significantly improved procedural safety and long-term outcomes. Therefore, the findings of earlier studies should be interpreted with caution, as they may not fully reflect current clinical practice.

OverallTherefore, although this meta-analysis provides strong evidence, the results should be interpreted with caution. Future research requires more high-quality prospective RCTs to validate the findings and optimize treatment strategies for WNAs.

## Conclusion

This study compared the efficacy and safety of SAC to NSAC in the treatment of WNAs and the results show that SAC demonstrated a significant advantage in complete occlusion rates during long-term follow-up, particularly in the treatment of RIA. However, the perioperative complication risk, especially ischemic events, was significantly higher with SAC, particularly when compared with CA. Also, there were no significant differences in other major outcomes although SAC was superior to BAC and DMC in terms of recurrence rates, indicating that SAC may not be the best choice for all patients. Therefore, treatment plans should be tailored to the specific circumstances of each patient. Future efforts should explore more precise treatment methods to improve efficacy and reduce risks, along with more high-quality research to validate these findings.

## Abbreviations

Aneurysmal subarachnoid hemorrhage: aSAH
Balloon-assisted coiling: BAC
Confidence intervals: CI
Conventional coiling: CA
Digital subtraction angiography: DSA
Dual microcatheter coiling: DMC
Flow diverters: FD
Glasgow Outcome Scale: GOS
Newcastle-Ottawa Scale: NOS
Non-stent-assisted coiling: NSAC
Modified Rankin Scale: mRS
Mean differences: MD
Unruptured intracranial aneurysms: UIA
Intracranial aneurysm: ICA
Propensity score matching: PSM
Ruptured intracranial aneurysms: RIA
Risk ratios: RR
Randomized controlled trials: RCTs
Stent-assisted coiling: SAC
Wide-neck intracranial aneurysms: WNAs
Woven endobridge: WEB
